# Evaluation of pathogenic bacteria growth and food quality during cold storage in Korean seasoning vegetables subjected to minimal processing

**DOI:** 10.1007/s10068-025-02002-x

**Published:** 2025-09-29

**Authors:** Areum Han, Yeonjin Woo, Sun-Young Lee

**Affiliations:** Department of Food and Nutrition, Chung-Ang University, 4726 Seodong-dearo, Anseong-si, Gyeonggi-do 17546 Republic of Korea

**Keywords:** Minimal processing, Microbiological growth, Food quality, Cold storage, Seasoning vegetables

## Abstract

Contamination and growth kinetics of pathogenic bacteria were evaluated in garlic, onion, and green onion subjected to different minimal processing at 10 °C for 15 days. Although, total aerobic bacteria (TAB) grew well in all processed vegetable samples (> 6 log CFU/g), maximum microbial population density (MPD) was significantly higher in chopped, sliced, or shredded than in peeled or cut vegetables. *Salmonella* spp. and *Listeria monocytogenes* growth kinetics paralleled TAB results. Moreover, vegetable samples that underwent complex processing showed a significant deterioration in visual quality and color. Therefore, the intensity of physical processing plays a pivotal role in promoting bacterial growth, with shredded or chopped processing posing the highest risk.

**Supplementary Information:**

The online version contains supplementary material available at 10.1007/s10068-025-02002-x.

## Introduction

Fresh-cut or minimally processed vegetables are known as products that have been subjected to minimal processing, such as trimming, washing, cutting, peeling, shredding, chopping, and/or drying (Ansah et al., 2018). Consumption of these vegetable products has consistently increased owing to the expansion of online market demand, an increase in single-person households, convenience, and efficiency (Jang et al., 2021; Kim et al., 2019). In general, these products are optimally packaged, stored, and distributed at an appropriate relative humidity and low temperature to protect their shelf life, physiological properties, and nutritional value (Ansah et al., 2018). Despite these efforts, microbiological contamination can occur pre-and post-harvest and during processing. In particular, freshly cut or pre-treated vegetables are highly susceptible to microbial contamination because intact plant tissues are wounded during processing and the released nutrients effectively nourish microbial growth (Jeddi et al., 2014).

Fresh-cut products host several human pathogenic bacteria, such as *Escherichia coli*, *Salmonella* spp., and *Listeria monocytogenes*, among others (Alfonzo et al., 2018). Therefore, foodborne disease outbreaks associated with fresh products have increased markedly worldwide in recent decades (Jeddi et al., 2014; Kim and Cheigh, 2022). Between 2010 and 2017, 12.7% of all cases of foodborne diseases in the United States were related to fresh produce (Carstens et al., 2019). Additionally, foodborne disease outbreaks associated with vegetables and fruits reportedly accounted for the highest proportion (19.8%) of such outbreaks in Korea from 1988 to 2016 (Park et al., 2017). In particular, *L. monocytogenes*, known to cause a high mortality rate, is reportedly the main pathogen detected in mushrooms, among agricultural products. However, several listeriosis outbreaks related to fresh produce have been reported in recent years, including corn salads in Italy (2000), mung bean sprouts in the USA (2014), and packaged salads in the USA (2016) (Alegbeleye et al., 2018; Wadamori et al., 2017). In addition, large-scale outbreaks of *Salmonella* spp., are a major concern for fresh produce safety. According to the Centers for Disease Control and Prevention (CDC) in the USA, from 1998 to 2012, *Salmonella* spp. ranked third among foodborne pathogens that caused food poisoning associated with fresh produce (Wadamori et al., 2017).

The fresh-cut produce market in Asia has grown rapidly, and minimally processed vegetables already account for a large share of the market in Thailand, Japan, and Korea (Uddin et al., 2021). Particularly, seasoning vegetables, such as garlic, onions, and green onions are widely consumed in Korea. They are a staple food in the Korean diet and are widely used as seasonings in various traditional dishes. Consequently, these products are processed, distributed, and consumed in diverse ways (Kim et al., 2020). Therefore, this study was conducted to evaluate the microbial contamination and market quality of garlic, onions, and green onions subjected to different minimal processes during cold storage.

## Materials and methods

### Preparation of minimally processed vegetables

Garlic heads, onions, and green onions were purchased at a local market in Anseong-si, Republic of Korea, on the day of the experiments, and stored at 10 °C until processing. Before use, all vegetable samples were washed separately under running tap water for approximately 30 s to remove organic materials, and the visibly damaged parts were discarded. The washed vegetable samples were then subjected to different minimal processes (peeling, cutting, slicing, shredding, and/or chopping), as these are the processing treatments mostly used in Korea. Knives, tongs, and cutting boards were disinfected with 70% ethanol before and after use.

### Total aerobic bacteria analysis of minimally processed vegetable samples

To evaluate total aerobic bacteria (TAB) in minimally processed vegetables during storage at 10 °C, 10 g of each sample were homogenized in 40 mL (1:5) of 0.85% saline solution using sterile filter stomacher bags (Difco Laboratories, Detroit, MI, USA) and stomached (BagMixer 400, Interscience, Breteche, France) for 1 min. One milliliter of each diluted sample was placed on a Petri film Aerobic Count Plate (3 M, Seoul, Korea) and incubated at 37 °C for 24 h.

### Growth kinetics of *Salmonella* spp. and *L. monocytogenes* on minimally processed vegetables

#### Preparation of pathogenic bacterial strains

Isolates of *Salmonella* spp. and *L. monocytogenes* obtained from vegetable products or foods containing vegetables were provided by the Korean Ministry of Food and Drug Safety (MFDS). Five *Salmonella* species were isolated from vegetable kimbap, bean sprouts, half-cooked carrots, pickled radishes, and iceberg lettuce. In turn, five *L. monocytogenes* strains were isolated from lettuce, kimbap cheese, green pudding salads, vegetable salads, and enoki mushrooms. All stock cultures were maintained at − 80 °C in 20% glycerol, and activated by culturing at 37 °C for 24 h in tryptic soy broth (TSB; Difco), in the case of *Salmonella* spp., or in TSB supplemented with 0.6% yeast extract (TSBY; Difco), in that of *L. monocytogenes*.

To prepare the inocula, each isolate of *Salmonella* spp. and *L. monocytogenes* was grown separately in 5 mL of TSB and TSBY, respectively, for 24 h at 37 °C. Overnight suspensions were centrifuged at 13,000×g for 3 min at 4 °C, washed once with sterile 0.1 M phosphate-buffered saline (PBS; pH 7.2), and resuspended in the same buffer. Five isolates of *Salmonella* spp., or *L. monocytogenes* were mixed in equal volumes in a microtube to form individual culture cocktails. Each bacterial mixture was then diluted using 0.2% peptone water (PW; Difco) to obtain approximately 5 log CFU/mL for bacterial growth kinetics experiments.

#### Bacterial growth in minimally processed vegetables

To evaluate the growth kinetics of *Salmonella* spp. and *L. monocytogenes* in minimally processed vegetables verified to be negative for both pathogens during storage at 10 °C, 0.1 mL of each bacterial mixture was inoculated on the vegetable surfaces (10 g) and the samples were dried in a laminar flow biosafety hood for at least 1 h. On each sampling day, 10 g of each sample were homogenized in 40 mL (1:5) of buffered peptone water (BPW) using sterile filter stomacher bags and stomached for 1 min. Aliquots (1 mL) were plated on four plates of xylose lysine deoxycholate agar (XLD; Difco) for *Salmonella* spp., or Oxford Agar Base (OAB; Difco) for *L. monocytogenes*. All agar plates were incubated at 37 °C for 24–48 h and developed colonies were counted.

### Prediction modeling

To obtain the primary prediction model by reliable estimation of the growth rate (GR) and lag time (LT), the survival curves of *Salmonella* spp. and *L. monocytogenes* present in minimally processed vegetables during cold storage were fitted to the modified Gompertz equation (Gibson et al., 1988) using Prism version 4.0 (GraphPad Software, San Diego, CA, USA).

1 $$ {\text{Y}} = {\text{N}}_{{\text{o}}} + {\text{C}} \times \exp \left[ { - \exp \left\{ {\left( {{2}.{718} \times \mu \, /{\text{ C}}} \right) \times \left( {{\text{lag }}{-}{\text{ X}}} \right)} \right\}} \right] $$

where, Y is the log-transformed cell number (log CFU/g); N_o_ is the log-initial cell number (log CFU/g); C is the difference between the initial and final cell numbers; X is the incubation time (d); μ is the maximum GR (1/d), and lag is the LT (d) before growth.

### Color analysis

The color characteristics of the minimally processed vegetable samples during cold storage were measured using a Minolta Color reader CR-20 (Minolta, Osaka, Japan). Color analysis was performed based on the average of five different measuring points for each sample, and expressed in terms of the CIE L*, a*, and b*, which are defined as lightness, red-greenness, and blue-yellowness, respectively. The total color difference (ΔE) and the browning index (*BI*) values were obtained using the values of CIE L*, a*, and b* (Liu et al., 2016).

2 $$ \Delta E = \sqrt {\left( {L* - L*_{0} } \right)^{2} + \left( {a* - a*_{0} } \right)^{2} + \left( {b* - b*_{0} } \right)^{2} } $$

where, L*_0_, a*_0_, and b*_0_ are the initial values for the minimally processed vegetable samples.

3 $$ BI = 100 \times \left( {x - 0.31} \right)/0.172 $$

where, $$x = {{\left( {a* + \, 1.75 \, \times L*} \right)} \mathord{\left/ {\vphantom {{\left( {a* + \, 1.75 \, \times L*} \right)} {\left( {5.645 \, \times L*} \right)}}} \right. \kern-0pt} {\left( {5.645 \, \times L*} \right)}} + \left( {*a - \left( {3.012 \, \times b*} \right)} \right)$$.

### Statistical analysis

Bacterial growth experiments were performed in triplicate, and the results were reported as means ± standard deviations. Statistically significant differences among groups (*p* < 0.05) were analyzed using ANOVA, followed by Duncan’s multiple range test (SAS Institute Inc., Cary, NC, USA).

## Results and discussion

### Bacterial growth in minimally processed vegetables during cold storage

**Fig. 1:**
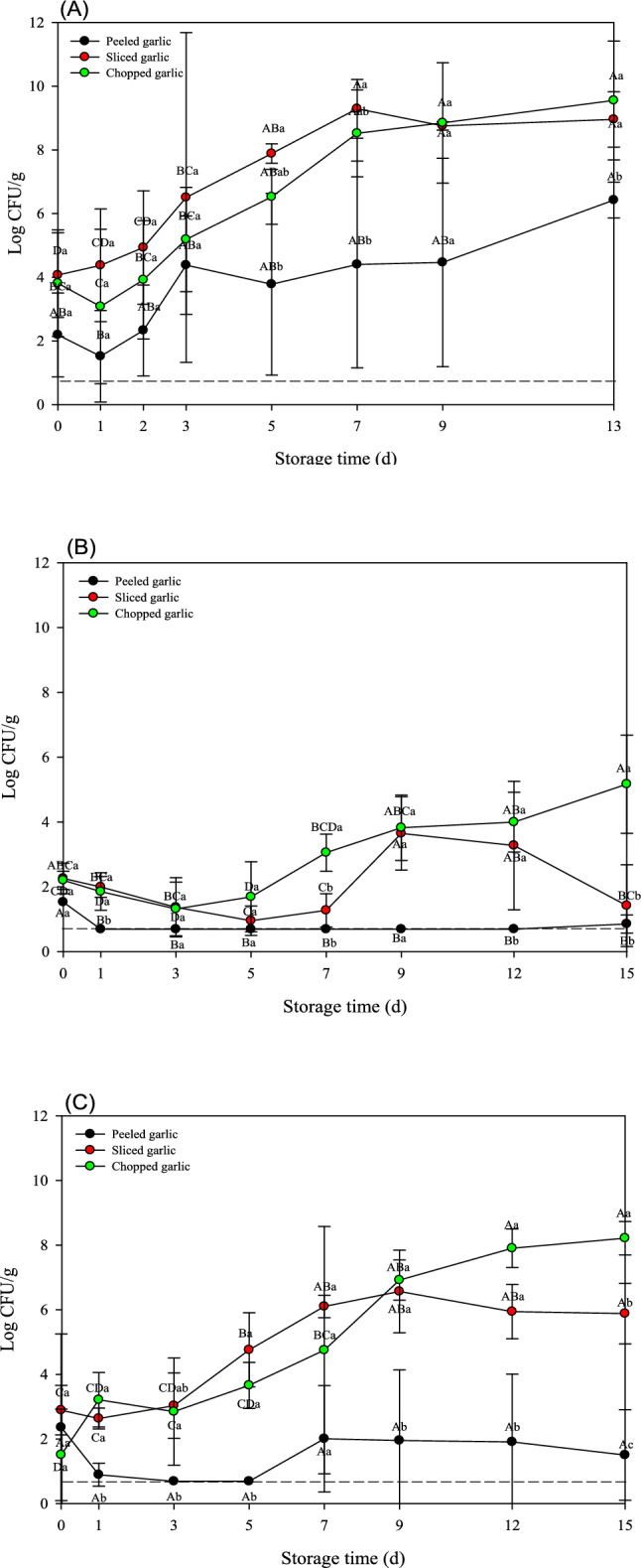
Populations (log CFU/g) of **A** total aerobic bacteria (TAB), **B** *Salmonella* spp., and **C** *L. monocytogenes* on minimally processed garlic samples during 13 or 15 days at 10 °C. *Different lowercase letters at the same time point indicate significant difference among treatments (*p* < 0.05). *Different capital letters within each sample indicate significant difference across time points (*p* < 0.05)

To investigate the effect of minimal processing on microbial growth during cold storage, each vegetable sample was subjected to various minimal processing methods, including peeling, slicing, shredding, cutting, or chopping. As shown in Fig. 1, TAB grew well in all garlic samples regardless of processing. Peeled garlic had slightly lower microbial counts than sliced or chopped garlic samples. Further, the TAB population in peeled, sliced, and chopped garlic gradually increased to 6.42 ± 0.56, 8.96 ± 0.87, and 9.55 ± 1.87 log CFU/g on the 13th day of storage, respectively (Fig. 1A). Moreover, microbial levels in sliced and chopped garlic increased significantly over time within the same processing treatment (*p* < 0.05). When *Salmonella* spp. and *L. monocytogenes* were inoculated into minimally processed garlic, both pathogens showed the greatest level of proliferation on chopped garlic, followed by those recorded for sliced or peeled garlic. Moreover, *L. monocytogenes* grew more than *Salmonella* spp. during cold storage (Fig. 1B and C).

**Fig. 2:**
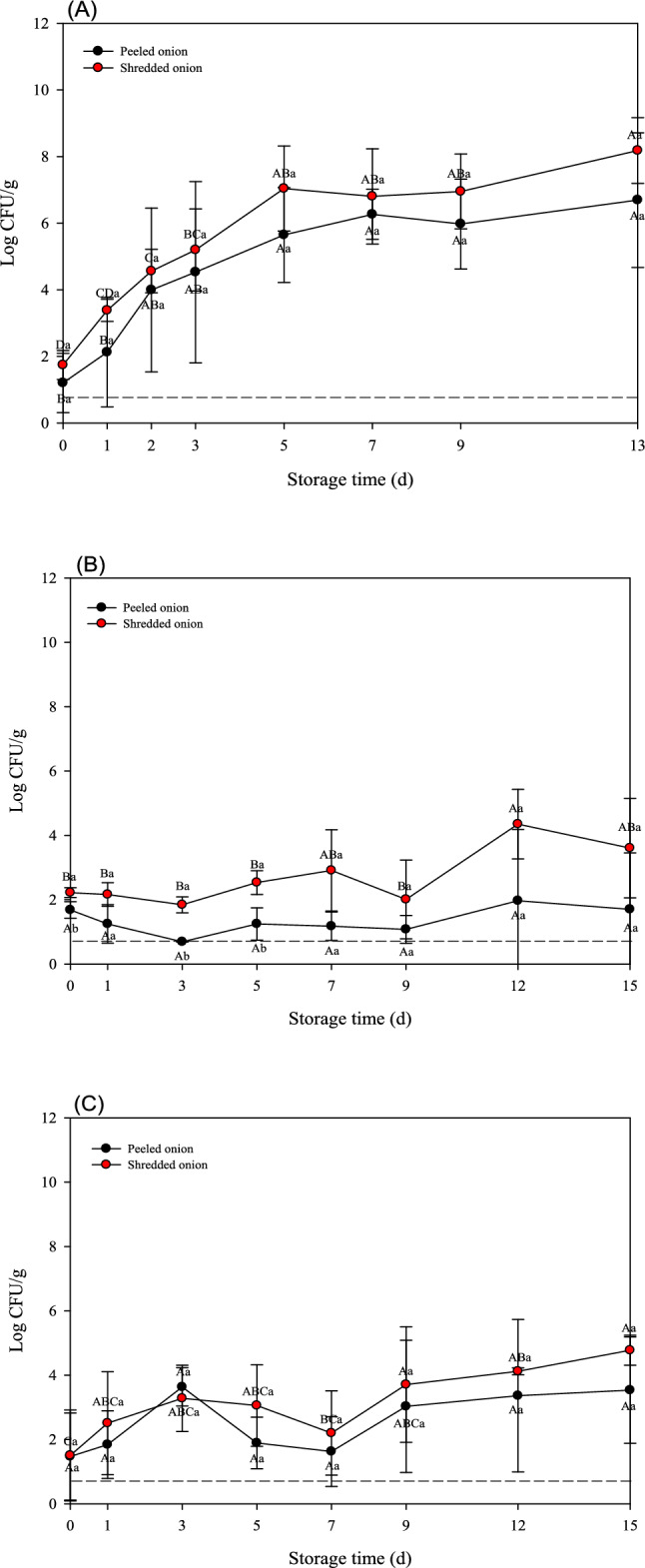
Populations (log CFU/g) of **A** total aerobic bacteria (TAB), **B** *Salmonella* spp., and **C** *L. monocytogenes* on minimally processed onion samples during 13 or 15 days at 10 °C. *Different lowercase letters at the same time point indicate significant difference among treatments (*p* < 0.05). *Different capital letters within each sample indicate significant difference across time points (*p* < 0.05)

Similarly, TAB for both minimally processed onions increased continuously, with shredded onions consistently showing higher microbial loads than peeled onions on day 2 and thereafter (Fig. 2A). *Salmonella* spp. and *L. monocytogenes* grew to 6.69 ± 2.02 and 8.18 ± 0.99 log CFU/g in peeled and shredded onions, respectively. Consistently, growth kinetics both pathogens in onions showed similar tendencies as those observed in garlic (Fig. 2B and C). The microbial loads of both pathogenic bacteria were higher in shredded than in peeled vegetable samples. Thus, in peeled and shredded onions, *Salmonella* spp. increased to 1.70 ± 1.75 and 3.60 ± 1.54 log CFU/g, respectively, while *L. monocytogenes* reached to 3.53 ± 1.65 and 4.77 ± 0.47 log CFU/g, respectively.

**Fig. 3:**
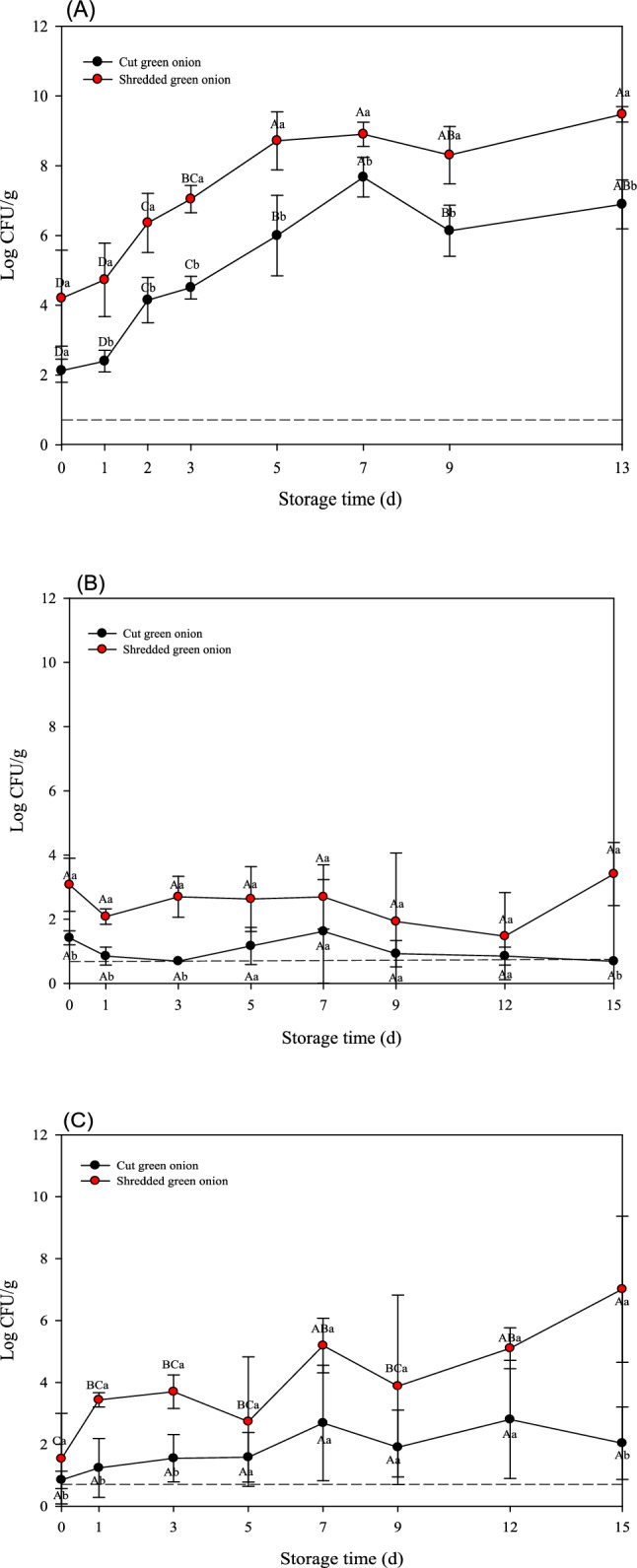
Populations (log CFU/g) of **A** total aerobic bacteria (TAB), **B** *Salmonella* spp., and **C** *L. monocytogenes* on minimally processed green onion samples during 13 or 15 days at 10 °C. *Different lowercase letters at the same time point indicate significant difference among treatments (*p* < 0.05). *Different capital letters within each sample indicate significant difference across time points (*p* < 0.05)

Similarly, total microbial levels increased steadily over time in both types of green onions (Fig. 3A). Further, shredded green onions showed consistently higher microbial growth than cut green onions throughout the storage period and they reached 6.89 ± 0.70 and 9.47 ± 0.22 log CFU/g in cut and shredded green onions, respectively, on the last day of storage. Additionally, similar growth behavior of *Salmonella* spp. and *L. monocytogenes* were observed in green onions to those observed in garlic and onion samples (Fig. 3B and C). For cut and shredded green onions, the number of *Salmonella* spp. on the 15th day of storage were 0.69 ± 0.00 and 3.41 ± 0.98 log CFU/g, respectively, while those of *L. monocytogenes* were 2.04 ± 1.17 and 7.01 ± 2.36 log CFU/g, respectively.

The microbial growth varied markedly among garlic, onion, and green onion samples stored at 10 °C. For garlic samples, all microbial populations (TAB, *Salmonella* spp., and *L. monocytogenes*) were significantly increased during storage (Fig. 1). In contrast, onion samples showed considerably lower microbial growth. Especially, both pathogenic bacteria remained at relatively low population levels without dramatic growth during storage (Fig. 2). Green onion samples exhibited an intermediate microbial growth between garlic and onion. While TAB exhibited a gradual increase similar to that observed in other vegetable samples, both pathogens eventually increased; but their maximum population were lower than those observed in garlic (Fig. 3).

Overall, these results indicate that microbial growth in minimally processed vegetables is dependent on the type of vegetables and processing methods. Nevertheless, mechanical processing such as shredding or chopping significantly accelerates microbial growth in fresh produce during cold storage. Consistently, Cui et al. (2022) showed that the number and growth rate of microbial communities in fresh broccoli were significantly higher after severe mechanical damage than those of fresh broccoli showing mild damage. These findings indicate a strong positive correlation between the degree of mechanical damage and microbial growth. According to Sun et al. (2022), washing, cutting, disinfection, and dehydration change the microbial community structure and dominant species of lettuce. In particular, after 3 days of storage, the total microbial load increased to more than 5 log CFU/g, and the relative abundance of *Pseudomonas* increased significantly, resulting in a noticeable decline in the quality of lettuce. Indeed, fresh-cut or processed vegetables degrade more quickly than whole produce because of the tissue damage caused by processing, which triggers various physical and physiological changes that affect produce viability and quality (Alfonzo et al., 2018). Specifically, during vegetable cutting, intracellular fluids may be released from the internal tissues and migrate to the surface. This mechanical damage provides a favorable environment for microbial growth, contributing to significantly higher microbial growth in processed vegetables than that in unprocessed products. Consequently, substantial microbial populations, including potential human pathogens, can colonize the surfaces of cut products (Raffo and Paoletti, 2022). Furthermore, several studies have shown that vegetable processing affects the effectiveness of washing and disinfection. Thus, for example, Nou and Luo (2010) investigated the efficacy of chlorinated water on cut romaine lettuce. Chlorine washing of intact lettuce leaves resulted in an approximately 1-log greater reduction in *E. coli* O157:H7 compared to that of cut leaves. Similarly, Hwang et al. (2017) investigated the effectiveness of chlorine- and alcohol-based disinfectants for pathogen reduction in whole or fresh vegetables, including Brussels sprouts, carrots, cherry tomatoes, bell peppers, and lettuce, among others. Interestingly, the sterilization effect was significantly lower for cut than for whole produce (*p* < 0.05).

### Modified Gompertz model for bacterial growth kinetics of minimally processed vegetables stored at 10 °C

Microbial growth parameters of the three minimally processed vegetable samples were analyzed using the modified Gompertz model (Table 1). The parameters assessed included: lag time (LT), growth rate (GR), and coefficient of determination (*R*^*2*^) for three microbial groups: TAB, *Salmonella* spp., and *L. monocytogenes.*

**Table 1:** Microbial growth parameters determined by the modified Gompertz model in minimally processed vegetables stored at 10 °C

Sample	TAB			*Salmonella* spp.			*L. monocytogenes*		
	LT (d)	GR (1/d)	*R*^*2*^	LT (d)	GR (1/d)	*R*^*2*^	LT (d)	GR (1/d)	*R*^*2*^
*Garlic*									
Peeled garlic	2.60	0.78	0.90	15.53	0.37	0.30	11.08	0.12	0.14
Sliced garlic	2.02	0.93	0.82	6.76	0.94	0.20	4.29	2.76	0.80
Chopped garlic	2.98	0.84	0.86	15.80	0.31	0.69	7.37	2.37	0.86
*Onion*									
Peeled onion	− 8.22	1.60	0.62	11.19	− 1.91	0.15	− 23.98	1.36	0.74
Shredded onion	− 28.39	23.52	0.83	11.86	0.36	0.71	− 52.01	3.11	0.52
*Green onion*									
Cut green onion	0.19	0.70	0.87	12.76	− 0.23	0.06	2.52	0.54	0.64
Shredded green onion	1.85	0.83	0.87	1.70	0.30	0.19	− 7.29	0.64	0.70

Total aerobic bacteria of all garlic samples showed positive LT values for the TAB range from 2.02 to 2.98 d, indicating a delay in the onset of microbial growth under cold conditions. Among all samples, sliced garlic showed the highest GR value (0.93 1/d), followed by chopped garlic (GR = 0.84 1/d), and peeled garlic (GR = 0.78 1/d). The model showed a good fit across all samples, with *R*^2^ values ranging from 0.82 to 0.90. These findings suggest that garlic slicing may provide a favorable environment for TAB growth. *Salmonella* spp. in sliced garlic showed the highest GR (0.94 1/d), despite having a relatively short LT (6.76 d). In contrast, both peeled and chopped garlic had significantly longer LT (15.53 and 15.80 d, respectively) and lower GR values (0.37 and 0.31 1/d, respectively). However, the model fit varied, with *R*^2^ values of 0.30 for peeled garlic, 0.20 for sliced garlic, and 0.69 for chopped garlic. These results indicate that, while *Salmonella* spp. may proliferate more rapidly in sliced garlic, the predictive confidence was the highest in the chopped garlic sample. The growth of *L. monocytogenes* varied significantly across garlic samples. *L. monocytogenes* proliferated most aggressively on processed garlic forms with greater surface exposure. Sliced garlic showed the most rapid bacterial growth (GR = 2.76 1/d), followed by those observed in chopped (GR = 2.37 1/d), whereas the lowest growth was observed in peeled garlic (GR = 0.12 1/d). The LTs followed an inverse trend, with peeled garlic showing the longest delay before the onset of growth (LT = 11.08 d). Indeed, *R*^2^ values indicated a strong model fit for chopped (0.86) and sliced garlic (0.80), but a poor fit for peeled garlic (0.14).

As for onions, TAB showed a negative LT for both onion samples. Thus, LT span for peeled and chopped onions were − 8.22 and − 28.39 d, respectively. This may suggest that early growth was rapid or, the existence of limitations to the model fit. In particular, shredded onion samples showed a remarkably high GR (23.52 1/d), which greatly exceeded that of peeled onion samples (GR = 1.60 1/d). These results strongly indicate that shredding onions provide a highly favorable environment for TAB growth by increasing the surface area for microbial attachment and damaging the onion tissues, hence facilitating microbial penetration. In the case of *Salmonella* spp., peeled onions showed a negative GR of − 1.91 1/d and an LT of 11.19 d, suggesting no growth under cold storage conditions. In contrast, microbial growth occurred in shredded onions with an LT of 11.86 d and a GR of 0.36 1/d. The *R*^2^ value was 0.15 for the peeled onion samples and 0.71 for shredded ones, indicating that the model fit the chopped vegetable material better. Similarly, shredded onions supported the most aggressive growth of *L. monocytogenes*, with a GR of 3.11 1/d, suggesting that shredded onions are more susceptible to *L. monocytogenes* contamination.

In turn, the TAB of cut onion samples showed an LT of 0.19 d and a GR of 0.70 1/d, while shredded green onion samples showed a slightly longer LT (1.85 d) and a similar GR (0.83 1/d). The model fit was close in both cases, with *R*^2^ values of 0.87 for both cut and shredded green onion samples. Both green onion types provided little support for the growth of *Salmonella* spp. Indeed, cut green onion samples showed a negative GR (− 0.23 1/d) and a short LT (12.76 d), suggesting that the growth of *Salmonella* spp. was inhibited under the cold storage conditions used in the experiments summarized herein. Similarly, shredded green onion supported a weak but positive GR (0.30 1/d) for these pathogens, with an LT of 1.70 d. The *R*^2^ values for both were low (0.06 for cut and 0.19 for shredded samples), indicating a poor model fit. Thus, *Salmonella* spp. did not thrive well in either form of green onion- experimental material tested herein. In contrast, *L. monocytogenes* showed modest growth in both types of green onion samples. The shredded samples supported a slightly higher growth of *L. monocytogenes* (GR = 0.64 1/d and LT = − 7.29 d) compared to the cut samples (GR = 0.54 1/d and LT = 2.52 d). Model fits were moderate, with *R*^2^ values of 0.70 and 0.64 for shredded and cut green onion samples, respectively. These results suggest that *L. monocytogenes* can grow under cold conditions, particularly in shredded vegetable materials.

Overall, the GR of TAB tended to increase with intensity of processing regardless of the vegetable material. On the other hand, little or no pathogenic bacterial growth was observed in vegetable materials minimally processed, although chopping and shredding did enhance microbial growth, if only slightly.

### Evaluation of food quality of minimally processed vegetables

**Fig. 4:**
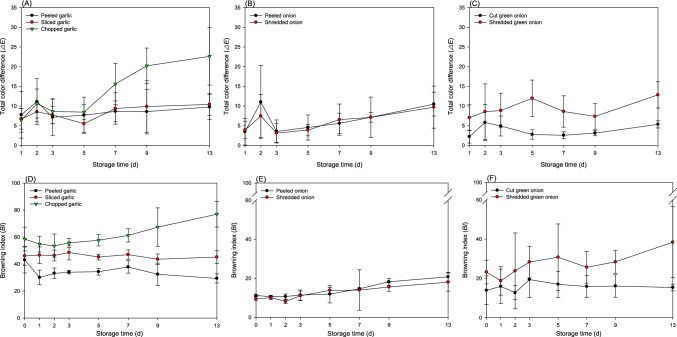
The change of **A–C** total color difference (Δ*Ε*) and **D–F** browning index (*BI*) of minimally processed (A and D) garlic, (B and E) onion, and (C and F) green onion samples during storage at 10 °C for 13 days

Color and appearance are important physical factors used to evaluate the overall market quality of fresh-cut or minimally processed vegetables during refrigeration. The effects of minimal processing on superficial color and appearance of vegetable samples stored at 10 °C for 13 days are shown in Table S1, Figs. 4, and S1. The lowest level of color change was observed in peeled garlic, compared with samples treated with other processing methods throughout the storage period. Furthermore, there was no significant difference in L* value (*p* > 0.05) among samples (Table S1). Specifically, L* and b* values of sliced and chopped samples significantly decreased with storage time, whereas a* values increased (*p* < 0.05). In general, Δ*Ε* values of minimally processed garlic gradually increased as storage time increased (Fig. 4A). In particular, a noticeable increase in Δ*Ε* value was reported in chopped garlic, with a final value of 22.6 ± 7.3 on the 13th day of storage (*p* < 0.05). Additionally, chopped garlic showed the highest *BI* value after the same time of storage among all the processing methods, followed by sliced and peeled garlic (Fig. 4D). As shown in Fig. S1, visual observation of simply peeled garlic barely changed over time; thus, produce appearance remained attractive. In contrast, chopped garlic changed color significantly from yellow to pale brown compared to garlic samples processed differently, making it visually unattractive to customers.

With respect to onions, the L* values of both sample types remained relatively constant throughout the storage period, indicating little color change in surface brightness over time (Table S1). However, when processing methods were compared, the L* value of peeled onions was higher than that of chopped onions during storage (*p* < 0.05). Consistently, both a* and b* values increased with storage time regardless of the processing method. Based on the changes in the color scale parameters (L*, a*, and b* values), Δ*Ε* and *BI* values of both onion sample types gradually increased with time of storage (Fig. 4B and E). In addition, visual observations showed that both minimally processed samples withered over time regardless of processing; however, the degree of wilting did not differ between the two onion sample types (Fig. S1).

A trend similar to that of the onion samples was observed for the L* values of the green onion samples (Table S1). The a* values of both green onion sample types increased significantly compared to those of the samples on the first day of storage (*p* < 0.05), indicating that their characteristic green color was lost over time. In particular, a* values of shredded green onions significantly changed from − 3.1 ± 0.7 to − 0.8 ± 0.6 during storage compared to simply cut green onions, which contributed to a large change in Δ*Ε* and *BI* values of shredded green onions (Fig. 4C and F). As for b* values, overall, shredded samples showed higher values than cut samples, but no significant change in the values of either sample type seemed associated with storage period (*p* > 0.05). Visual observations revealed that shredded green onions lost moisture and turned brown, making them less attractive than cut green onions (Fig. S1).

Visual parameters, including color, shape, physical changes, and wilting, can provide indirect information about freshness, microbial spoilage, and vegetable quality, which in turn influences consumer preference, acceptability, and marketability (Alfonzo et al., 2018). Therefore, the greatest challenge in producing minimally processed vegetables is preserving the original quality of the fresh product. Physical damage to vegetable tissues induces ethylene production, which in turn stimulates enzymes responsible for physiological and biochemical changes leading to faster ripening and decay as in senescence. In addition, ethylene triggers the metabolism of phenolic compounds and enzymatic browning, potentially leading to sensory alterations (Schuh et al., 2019). Sun et al. (2022) reported that processing affects quality changes in texture, appearance, flavor, and color. Thus, for example, cut lettuce showed a decline in food quality during storage, including loss of firmness, discoloration, and an increasingly foul odor. Also, vegetable quality decline is closely related to changes in contamination with microbial communities. In particular, the proliferation of spoilage-related bacteria, such as *Pseudomonas* and *Erwinia*, has a significant impact on tissue degradation and sensory defects. Additionally, Alfonzo et al. (2018) reported that cutting of red chicory during cold storage had negative effects on weight loss, titratable acidity (TA), and leaf color. In particular, the redness (a*) and hue angle of cut red chicory were significantly reduced compared to those recorded for intact leaves.

Therefore, the degree of physical processing is an important factor affecting microbial growth on seasoning vegetables such as garlic, onion, and green onion. Although all vegetables are susceptible to microbial contamination over time, highly processed vegetables are more likely to show significantly increased growth of microorganisms, particularly TAB and *L. monocytogenes*. In contrast, minimum processing (e.g., peeling or cutting) entails greater resilience to microbial contamination and is better suited for safe cold storage. Moreover, food quality, especially with regard to appearance and color, is negatively affected by the degree of physical processing and shows greater deterioration with increasing processing intensity. These observations highlight the importance of stricter hygienic control, improved storage management, and potentially shorter shelf-life for intensely processed vegetable products.

## Supplementary Information

Below is the link to the electronic supplementary material.

Supplementary file1 (DOCX 1201 KB)
