# Supplementary material for: Evaluation of pathogenic bacteria growth and food quality during cold storage in Korean seasoning vegetables subjected to minimal processing
Source: Food Sci Biotechnol. 2025 Sep 29;34(16):4059–67. doi: 10.1007/s10068-025-02002-x (PMC12589684; doi:10.1007/s10068-025-02002-x)
Supplement: Supplementary file 1 — Supplementary file1 (DOCX 1201 KB) [file 10068_2025_2002_MOESM1_ESM.docx]

**Supplementary Figure**

**Supplementary Figure 1.** Changes in the visual quality of minimally processed vegetables during storage at 10°C for 13 days

| Sample | | Storage (d) | | | | | | | |
| --- | --- | --- | --- | --- | --- | --- | --- | --- | --- |
|  |  | 0 | 1 | 2 | 3 | 5 | 7 | 9 | 13 |
| Garlic | Peeled garlic | 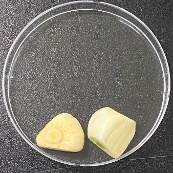 | 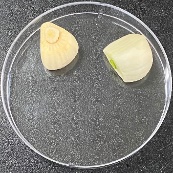 | 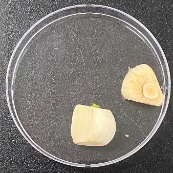 | 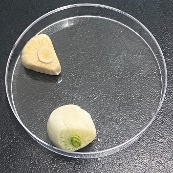 | 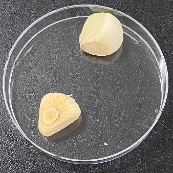 | 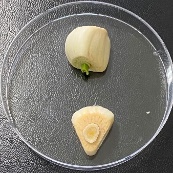 | 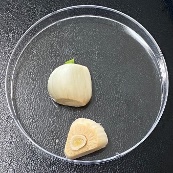 | 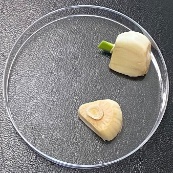 |
|  | Sliced garlic | 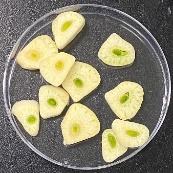 | 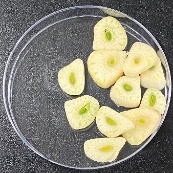 | 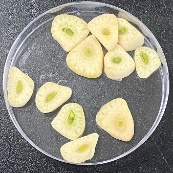 | 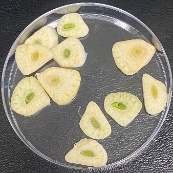 | 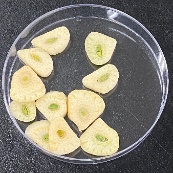 | 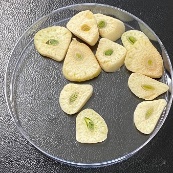 | 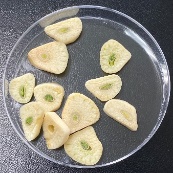 | 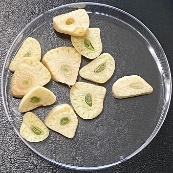 |
|  | Chopped garlic | 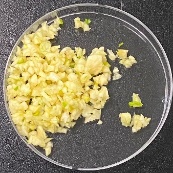 | 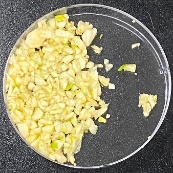 | 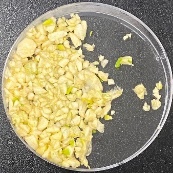 | 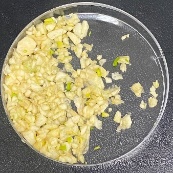 | 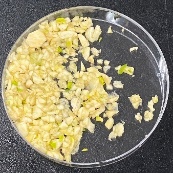 | 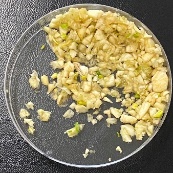 | 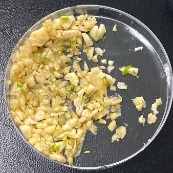 | 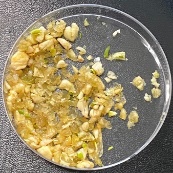 |
| Onion | Peeled onion | 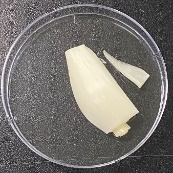 | 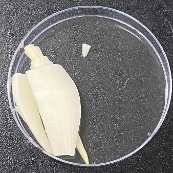 | 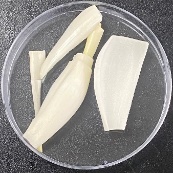 | 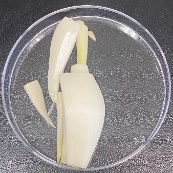 | 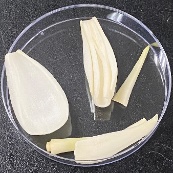 | 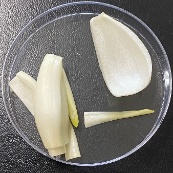 | 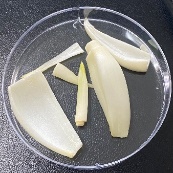 | 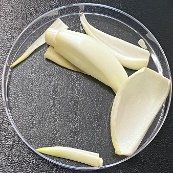 |
|  | Shredded onion | 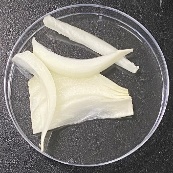 | 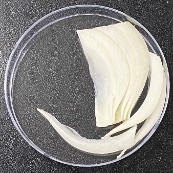 | 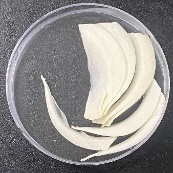 | 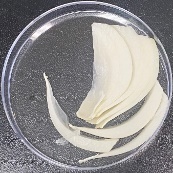 | 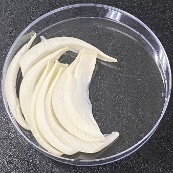 | 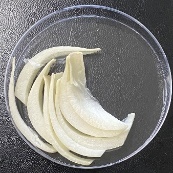 | 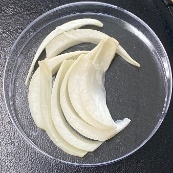 | 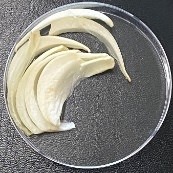 |
| Green onion | Cut green onion | 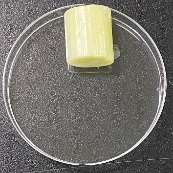 | 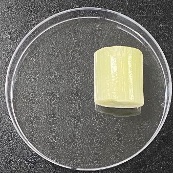 | 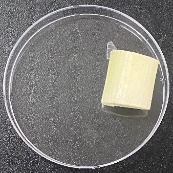 | 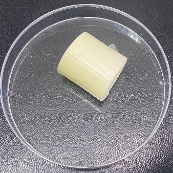 | 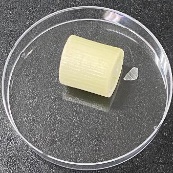 | 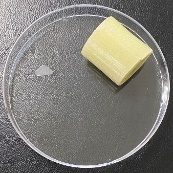 | 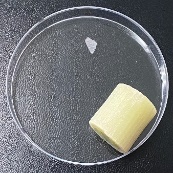 | 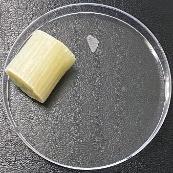 |
|  | Shredded  green onion | 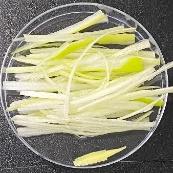 | 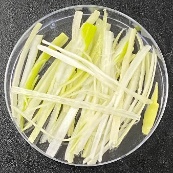 | 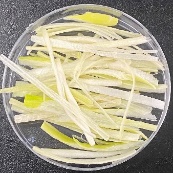 | 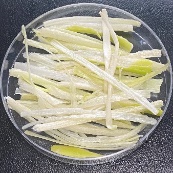 | 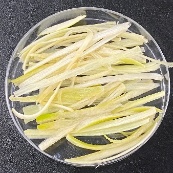 | 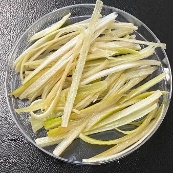 | 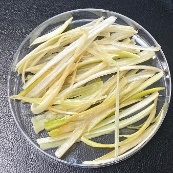 | 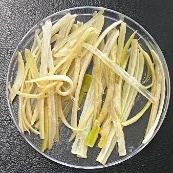 |

**Supplementary Figure 1.**

**Supplementary Table 1.** Color parameters for minimally processed vegetables during storage at 10°C for 13 days

| Sample | | Storage (d) | *L** | *a** | *b** |
| --- | --- | --- | --- | --- | --- |
| Garlic | Peeled garlic | 0 | 77.3±4.6Ba | 2.3±1.2Aa | 25.9±3.2Ab |
|  |  | 1 | 80.9±3.6ABa | 0.3±1.1Ca | 21.2±3.5CDb |
|  |  | 2 | 79.0±8.0ABa | 1.3±1.9ABCa | 21.8±2.5BCDb |
|  |  | 3 | 82.9±0.5Aa | 0.6±0.5Ca | 23.9±0.7ABCc |
|  |  | 5 | 82.6±1.5Aa | 1.3±0.9ABCb | 23.7±0.8ABCb |
|  |  | 7 | 80.5±3.2ABa | 2.0±1.6ABa | 24.6±1.2ABa |
|  |  | 9 | 79.7±4.3ABa | 0.9±1.2BCb | 22.0±5.3BCDa |
|  |  | 13 | 77.7±4.0Ba | 0.6±0.6Cb | 19.8±2.5Db |
|  | Sliced garlic | 0 | 74.6±3.1ABa | -1.4±1.1Dc | 28.6±2.5Aa |
|  |  | 1 | 74.3±5.1ABb | -0.6±0.6Db | 28.4±2.1Aa |
|  |  | 2 | 72.3±5.8ABCb | 0.5±0.2Cab | 27.0±2.3Aba |
|  |  | 3 | 74.7±4.6ABb | 1.9±2.5Ba | 28.2±0.8Aa |
|  |  | 5 | 75.7±2.2Ab | 1.7±0.2Bab | 27.2±0.5ABa |
|  |  | 7 | 70.2±6.0BCb | 1.8±0.5Ba | 25.8±1.1Ba |
|  |  | 9 | 70.8±8.0ABCb | 2.5±0.8ABa | 24.0±2.0Ca |
|  |  | 13 | 68.5±5.3Cb | 3.2±1.1Aa | 23.7±2.3Ca |
|  | Chopped garlic | 0 | 69.5±2.1Ab | 1.2±0.6CDb | 30.8±2.9Aa |
|  |  | 1 | 64.8±1.8Bb | -0.1±0.8Eab | 28.0±2.3Ba |
|  |  | 2 | 60.9±4.5Bc | -0.3±0.9Eb | 25.7±2.8CDEa |
|  |  | 3 | 62.6±2.6Bc | 0.8±0.7Da | 26.9±1.3BCDb |
|  |  | 5 | 62.7±2.5Bc | 1.9±0.4BCa | 27.2±0.8BCa |
|  |  | 7 | 55.7±4.6Cc | 2.5±0.6Ba | 24.9±1.9DEa |
|  |  | 9 | 50.9±4.8Dc | 3.3±1.0Aa | 23.7±2.4Ea |
|  |  | 13 | 48.1±7.6Dc | 3.3±0.9Aa | 25.0±2.9DEa |
| Onion | Peeled onion | 0 | 80.4±4.0Aa | -2.2±0.4Aa | 10.1±0.4CDa |
|  |  | 1 | 81.8±2.2Aa | -2.2±0.5Aa | 9.8±0.5CDa |
|  |  | 2 | 70.4±8.1Ca | -2.0±0.6Ab | 8.6±1.7Da |
|  |  | 3 | 82.2±3.4Aa | -2.3±0.8Ab | 10.6±2.5Ca |
|  |  | 5 | 81.5±1.5Aa | -3.2±0.3Bb | 11.3±3.4Ca |
|  |  | 7 | 81.2±1.4Aa | -3.8±0.3Cb | 13.7±0.7Ba |
|  |  | 9 | 80.7±2.7Aa | -4.3±0.2Db | 16.4±1.6Aa |
|  |  | 13 | 74.9±3.1Ba | -5.0±0.3Eb | 17.4±1.4Aa |
|  | Shredded onion | 0 | 70.1±5.0ABb | -2.0±0.2BCDa | 7.8±1.1BCb |
|  |  | 1 | 72.2±6.0Ab | -2.1±0.1Da | 8.4±1.2BCb |
|  |  | 2 | 64.3±4.8Ba | -1.4±0.4ABa | 6.1±1.2Cb |
|  |  | 3 | 72.6±4.9Ab | -1.3±1.1Aa | 8.6±1.6BCb |
|  |  | 5 | 70.2±3.4ABb | -1.5±0.5ABCa | 10.2±1.0ABa |
|  |  | 7 | 69.5±4.8ABb | -1.8±0.4ABCDa | 10.2±7.5ABa |
|  |  | 9 | 65.2±10.1Bb | -2.0±0.4CDa | 10.8±1.8ABb |
|  |  | 13 | 64.7±7.0Bb | -1.3±0.7Aa | 11.7±2.9Ab |
| Green onion | Cut green onion | 0 | 77.2±3.6ABa | -2.2±1.2BCa | 11.2±5.3Aa |
|  |  | 1 | 76.2±4.4ABa | -2.3±1.3Ca | 12.3±6.0Aa |
|  |  | 2 | 73.2±7.1Ba | -1.5±0.5ABCa | 9.6±2.0Aa |
|  |  | 3 | 73.8±5.9ABa | -1.6±0.7ABCa | 13.6±5.2Aa |
|  |  | 5 | 76.1±4.7ABa | -1.9±1.0BCa | 12.9±4.2Aa |
|  |  | 7 | 77.6±4.4ABa | -1.6±0.6ABCa | 12.3±3.5Aa |
|  |  | 9 | 78.3±3.2Aa | -1.4±0.5ABa | 12.5±3.7Ab |
|  |  | 13 | 76.6±5.1ABa | -1.0±0.1Aa | 11.6±0.5Ab |
|  | Shredded green onion | 0 | 59.3±5.9BCDb | -3.1±0.7Da | 14.2±3.3ABa |
|  |  | 1 | 62.4±3.6ABb | -2.5±0.8CDa | 12.2±3.7Ba |
|  |  | 2 | 55.5±7.4CDb | -2.4±1.5CDb | 12.5±7.7ABa |
|  |  | 3 | 64.7±1.5Ab | -2.0±0.8BCa | 17.2±3.8Aa |
|  |  | 5 | 61.7±5.6ABb | -1.3±0.4ABa | 16.2±6.3ABa |
|  |  | 7 | 60.8±6.0ABCb | -1.0±1.0Aa | 14.1±2.6ABa |
|  |  | 9 | 60.6±3.8ABCb | -0.9±0.7Aa | 15.6±2.3ABa |
|  |  | 13 | 55.0±7.0Db | -0.8±0.6Aa | 17.2±5.3Aa |

Values expressed as the mean ± standard deviation.

Different lowercase letters indicate significant differences among differently processed vegetable samples at the same storage time point (*p* < 0.05).

Different capital letters indicate significant differences among storage periods within the same treatment of each vegetable sample (*p* < 0.05).
